# Enhanced You Only Look Once X for surface defect detection of strip steel

**DOI:** 10.3389/fnbot.2022.1042780

**Published:** 2022-11-21

**Authors:** Ruiqi Wu, Feng Zhou, Nan Li, Haibo Liu, Naihong Guo, Rugang Wang

**Affiliations:** ^1^School of Information Technology, Yancheng Institute of Technology, Yancheng, China; ^2^Yancheng Xiongying Precision Machinery Company Limited, Yancheng, China

**Keywords:** strip steel, surface defect detection, YOLOX, lightweight, attention module

## Abstract

Using deep learning-based methods to detect surface defects in strip steel can reduce the impact of human factors and lower costs while maintaining accuracy and efficiency. However, the main disadvantages of this method is the inability to tradeoff accuracy and efficiency. In addition, the low proportion of valid information and the lack of distinctive features result in a high rate of missed detection of small objects. In this paper, we propose a lightweight YOLOX surface defect detection network and introduce the Multi-scale Feature Fusion Attention Module (MFFAM). Lightweight CSP structures are used to optimize the backbone of the original network. MFFAM uses different scales of receptive fields for feature maps of different resolutions, after which features are fused and passed into the spatial and channel attention modules in parallel. Experimental results show that lightweight CSP structures can improve the detection frame rate without compromising accuracy. MFFAM can significantly improve the detection accuracy of small objects. Compared with the initial YOLOX, the mAP and FPS were 81.21% and 82.87Hz, respectively, which was an improvement of 4.29% and 12.72Hz. Compared with existing methods, the proposed model has superior performance and practicality, verifying the effectiveness of the optimization method.

## Introduction

Strip steel is used in a fairly wide range of applications and is in high demand in industrial production. Its surface quality is one of the most important criteria for the quality of strip steel. In recent years there has been an increasing interest in Automated Surface Inspection Systems (ASIS) based on machine vision. ASIS is a non-contact, non-destructive, and fully automated solution to complement or replace conventional detectors (Kapsalas et al., 2007; Ghorai et al., 2013; Xie et al., 2014; Kuo et al., 2018). To reduce labor costs and improve detection efficiency, deep learning-based detection methods replace traditional methods (Jia et al., 2004; Amid et al., 2012; Jeon et al., 2014; Tikhe, 2014; Yuan et al., 2015). Deep learning techniques are becoming more and more important in solving many challenging computer vision tasks, such as urban traffic (Ali et al., 2019, 2021a,b), multi-object detection (Liu et al., 2020), and medical image segmentation (Zhao et al., 2019; Hesamian et al., 2020). All features are extracted automatically in the multi-layer structure of a deep neural network. Approaches based on deep neural network have better detection performance compared to manual methods. There are two types of deep learning-based object detection methods: two-stage and one-stage. The two-stage network is divided into two steps, region proposal, and image classification, and has a high detection accuracy. Specifically, the commonly used two-stage object detection methods include R-CNN (Girshick et al., 2014), Fast R-CNN (Girshick, 2015), Faster R-CNN (Ren et al., 2017), SPP-Net (He et al., 2015), etc. On the other hand, the one-stage model performs classification and regression directly. Thus, this method is fast in detection but low in accuracy, especially for overlapping objects and small objects (Li K. et al., 2022). Specifically, the commonly used single-stage object detection methods include YOLO (Redmon et al., 2016; Redmon and Farhadi, 2017, 2018; Bochkovskiy et al., 2020; Ge et al., 2021) and SSD (Liu et al., 2015).

The surface defect detection task is foreground localization and identification against a simple background. As productivity increases, production speeds increase and defect areas decrease, inspection equipment places greater demands on the embedded algorithms. During conventional production, strip steel is rolled at speeds of up to 20m/s or more (Su and Luo, 2022) and in widths of up to 1 meter. Such high-speed, real-time operation requires special image processing equipment and software. Although the two-stage network has a high level of inspection accuracy, it is far from being fast enough to meet the requirements of high-speed rolling lines. One-stage networks, although fast in detection, tend to be less accurate, especially for small and stacked objects. The major objective of this paper is to improve the speed and accuracy of the detection of surface defects in strip steel. A lightweight YOLOX surface defect detection method for strip steel combined with a multi-scale feature fusion attention module is proposed. The method enables high-speed, high-performance models to be deployed in the production process, enabling unmanned, fast, and robust localization and classification of strip steel surface defects. The two main contributions of this method are as follows.

(1) Lightweight network architecture: Lightweight design of the backbone network. A lightweight CSP structure is proposed: firstly, the number of channels is compressed by Bottleneck, and secondly, the normal K*K convolutional filters for feature extraction are replaced by K*1 and 1*K depthwise separable convolution filters. This structure reduces the size of the model, the number of parameters, and the number of calculations, and increases the speed of detection, without compromising the accuracy of the detection.
(2) New attention module: A multi-scale feature fusion attention module is proposed. The characterization of the underlying features is enhanced by applying different scale receptive fields and feature fusion to feature maps of different resolutions passed into the Neck from the backbone network. The features are then passed into the spatial-channel attention module in parallel to avoid the two modules influencing each other. The module significantly improves the accuracy of small-area defect detection.

## Related work

The detection of surface defects is an important process of the strip steel production process. With the development of deep learning, researchers have introduced convolutional neural networks into strip steel surface defect detection methods and continue to improve detection accuracy in their research. Based on the single-stage YOLOV5 model, Li and Wang (2021) selected the best model by transfer learning and group comparison. 77.3% of mAP values were achieved on the NEU-DET dataset. Xing and Jia (2021) proposed a convolution network classification model with symmetric modules to extract features and designed an optimized IOU (XIoU). The result shows that their model achieves 79.89% mAP on NEU-DET and 78.44% mAP on the self-made detection dataset. Cheng and Yu (2020) proposed RetinaNet with difference channel attention and adaptively spatial feature fusion. Results showed that the new network achieved 78.25 mAP and improved by 2.92% over RetinaNet. Han et al. (2021) proposed a Faster R-CNN detection method with feature fusion and cascade detection. Feature fusion and cascading of detection networks improved detection accuracy by 11.86% and 2.37%, respectively. The detection and accurate positioning of defects on metal surfaces are the basis for automated manufacturing technology. Sun et al. (2022) added a frequency domain channel attention module to YOLOV5 and increased the mAP from 79.9 to 85.5%, keeping the FPS at 27.71. However, the most difficult type of crazing to detect in the NEU-DET dataset was omitted from the paper because the image defects were not obvious, so the effectiveness of the attention module in detecting all types of defects is not known. Yuan et al. (2022) made a series of small improvements to ResNet18 and were able to achieve an accuracy of 97.11% when acting together. Liang et al. (2022) proposed a deep residual shrinkage network model. The accuracy was improved by optimizing the Adam and activation functions and was able to achieve 98.88% on the test set. The attention module was introduced and optimized on ResNet 50 and 101 by Lu et al. (2021) and preprocessed the dataset by false color enhancement. The optimal model was able to achieve 98.05% accuracy with an FPS of 7.69 on the NEU-DET dataset. Liu Y. et al. (2021) replaced the backbone network of YOLOv4 with MobileNetv3 to improve the detection frame rate. And the problem of poor detection caused by uneven positive and negative samples was addressed by redefining the confidence loss. The mAP and FPS of the final model were 91.13% and 26.39%, respectively.

In response to production demands, a large and growing number of researchers have investigated how to increase the speed of detection. Almost all of these studies are based on one-stage object detection networks. Xu et al. (2021) fused the shallow features of DarkNet53 layer 11, the backbone network of YOLOV3, with the deep features to generate a new feature layer. The experimental results show that the improved YOLOV3 has an mAP of 75.1%, which is highly accurate in locating small defect objects while having some real-time performance. Liu Y. et al. (2022) used the same idea to improve YOLOV3. Adding 1 layer of prediction scale to the 3-layer prediction scale of the backbone network DarkNet53, and then densely connecting the multi-scale feature maps across layers, enhances the ability to characterize dense minute defects. The method has an average detection accuracy of 89.24% and can detect 25.62 images of 416*416 pixels per second. Li X. et al. (2022) achieved a lightweight model by replacing the backbone network of YOLOV5, reducing the size by 10.4%; and introducing an attention module to improve the detection accuracy by 3.3%.

Small object detection has long been a difficult and hot topic in computer vision (Tong et al., 2020). Han and Li (2022) proposed the method of increasing residual connections and cross-layer attention to improving the detection capability of the model for small objects in remote sensing images. This method verified that the bottom feature map and attention module in the feature pyramid are very important to improve the performance of small object detection. Liu X. et al. (2022) proposed an improved TTB-SSD (Top to Bottom SSD) algorithm combining PANet multi-scale feature fusion network and top-down feature fusion path. This method allows for more accurate localization of small objects and increases the accuracy of small object detection. Bosquet et al. (2021) proposed an end-to-end spatio-temporal convolutional neural network for small object detection in video. This architecture detects small objects over time and correlates pairs of the top-ranked regions with the highest likelihood of containing those small objects. With the development of newer production lines, the size and distinctiveness of the defects are being reduced. The challenge of small object detection is beginning to affect the field of surface defect detection. Zhang et al. (2022) added three feature extraction layers and three small-sized anchor frames to the backbone network to address the situation of the non-detection of fine defects. The algorithm showed a large improvement in recall and overall accuracy. Liu K. et al. (2021) proposed an end-to-end defect detection framework based on the Adaptive Sort Fusion Attention Network. The method can extract features at different scales and levels for different defects, while suppressing the background, allowing better extraction of features for small objects. The method improves detection accuracy, especially for small-area objects. The mAP is improved to 83.2%.

## Enhanced YOLOX for surface defect detection of strip steel

### Backbone

#### The YOLOX object detection network

**FIGURE 1:**
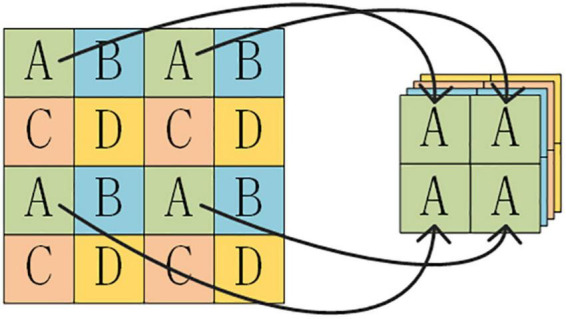
Focus structure.

The YOLO series is a typical one-stage object detection method. During the prediction process, the input image is divided into a grid of three resolutions, which are used to predict small, medium, and large objects. Several prior bounding boxes exist for each feature point. The prediction result of the network makes a judgment on whether the interior of the prior bounding box contains an object or not and adjusts the prior bounding box to obtain a prediction bounding box. YOLOX is based on YOLOv3-Darknet53, incorporating relevant technologies from YOLOv5 and optimized for improvement. In the backbone network, YOLOX uses the Focus structure to divide and stack feature point information onto the channels. The Focus structure is shown in Figure 1.

YOLOX uses the SiLU activation. The SiLU activation has the properties of being upper bound-free with lower sessions, smooth and non-monotonic. SiLU outperforms ReLU on deep models, which is defined as follows:


(1) SiLU(x)=x∗sigmoid(x)

YOLOV3, YOLOV4, and YOLOV5 are all Anchor-Based, while YOLOX uses the Anchor-Free approach. Anchor-based object detection networks cluster *a priori* frames when predicting, which has a high time complexity and requires different anchor frames to be designed for different datasets, limiting applicability. The anchor-free object detection network, on the other hand, only requires regression on centroid coordinates and width and height, which is of low time complexity, but its accuracy is not up to that of the anchor-based detection network. The past YOLO series implemented classification and regression inside a 1*1 convolution, an approach that YOLOX felt would have a detrimental effect on the network. Therefore, the decoupling head is divided into two parts in YOLOX. Classification and regression are implemented separately, and the two parts of information are then integrated when making predictions. The *reg* branch calculates the loss through the IoU of all ground truth and prediction, as shown in the following equation.


(2) $$L_{reg}=-log(IoU(B_{gt},B_{pred}))$$

The *cls* and *obj* branches are trained by the BCE loss function, as shown in the following equation.


(3) $$L_{cls}=-\sum_{i=1}^{n}\left(t_{i}logp_{i}+(1-t_{i})log(1-p_{i})\right)$$

**FIGURE 2:**
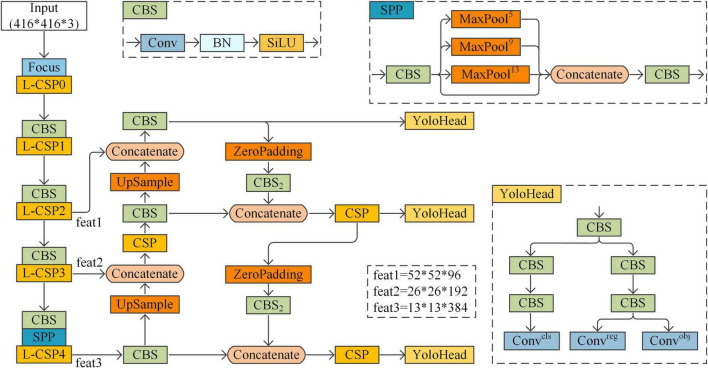
Enhanced YOLOX structure.

In this paper, the YOLOX target detection model will be enhanced for application to the task of detecting surface defects in strip steel. The structure of the enhanced YOLOX model is shown in Figure 2.

#### Lightweight modules

**FIGURE 3:**
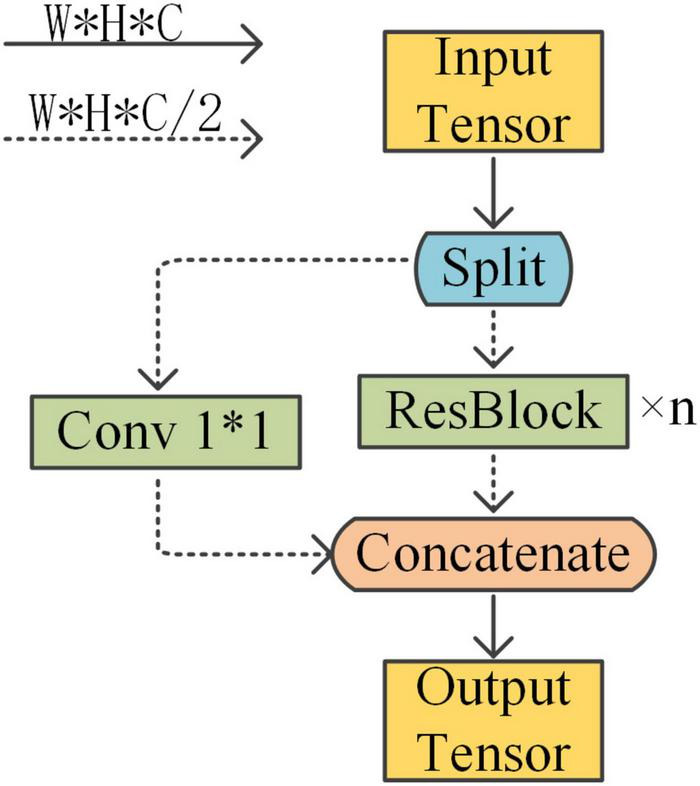
Applying CSP to ResBlock.

The backbone feature extraction network used by YOLOX is Darknet53, on which this paper uses CSPDarknet53 (Cross Stage Partial) as the backbone and designs the residual block in the backbone to be lighter. CSPDarknet53 is the backbone network used by YOLOv4. It is a backbone designed on the basis of the YOLOv3 backbone network Darknet53, drawing on the experience of CSPNet 2019. CSPDarknet gains a better learning advantage through the overlay of CSP structures and residual blocks. This cross-stage local network maximizes the difference in gradient union as a way to avoid different convolutional layers learning the same gradient information. There is a residual structure of repeated calls in the CSP structure and the parameters of the model are mainly concentrated in this part. The structure of the CSP applied to the residual block is shown in Figure 3.

**FIGURE 4:**
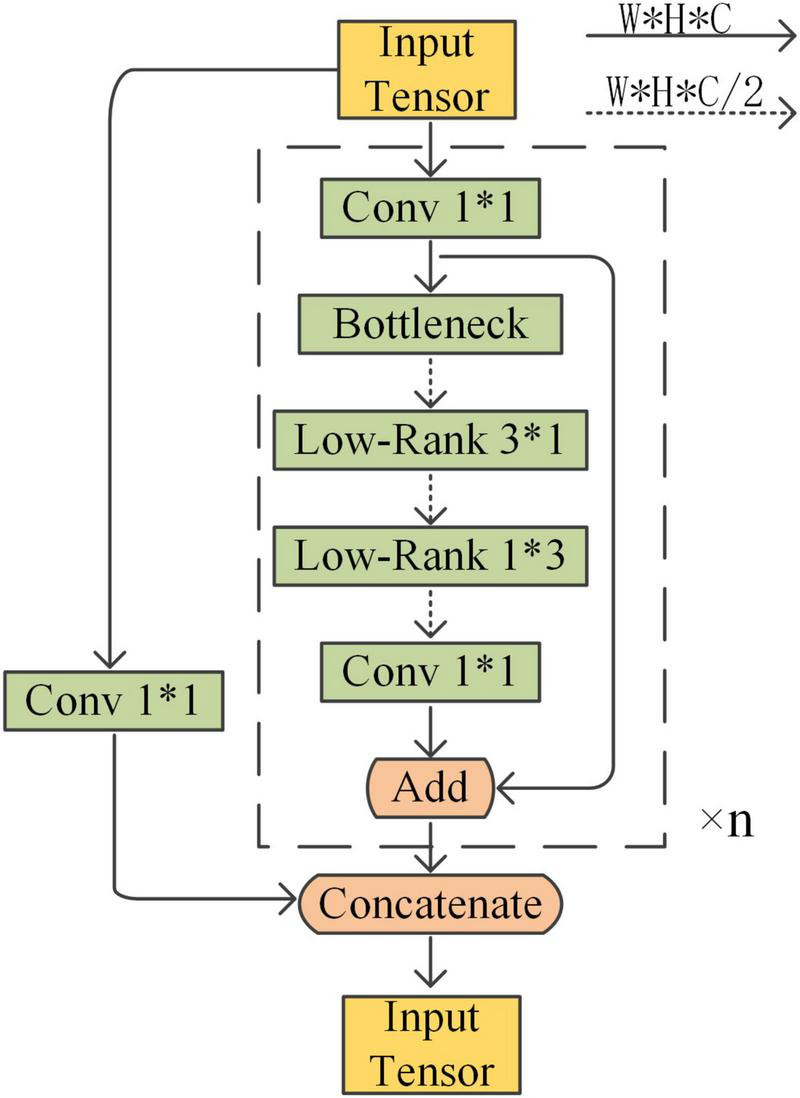
L-CSP structure.

To reduce the number of parameters and computation without affecting the feature extraction performance, an L-CSP structure is proposed in this paper. In the residual block of the basic module, a layer of Conv2D is used as the compression bottleneck to reduce the number of channels. The low-rank decomposition is also used at the filter level, which means decomposing one K*K filter into two filters of size K*1 and 1*K and coordinating more weight information into lower-rank filters. In this paper, the 3*3 ordinary Conv2D after the bottleneck is replaced with 3*1 and 1*3 depth-separable convolution to further improve the model operation speed. Finally, the number of channels is adjusted with a layer of convolution and the features are fused and output. The L-CSP structure is shown in Figure 4.

In the five CSP modules of the original CSPDarknet53, the residual structures were stacked one, two, eight, eight, and four times, for a total of 52 convolutional layers. To improve the speed of the model, the number of stacks was changed to 1, 1, 3, 3, and 1; the number of output channels of the last four CSP modules was scaled to 0.375 times the original number, 48, 96, 192 and 384, respectively. The CBS module with a stride of 2 acts as a downsampler for the network.

### Multi-scale feature fusion attention module

Based on the detection results of the unoptimized model, the detection of Crazing was the least satisfactory of all the object species, with very low AP values. This type of object has a small relative area and is a small object. To improve the detection accuracy of the model for small objects, this paper proposes a new attention module, the Multi-scale Feature Fusion Attention Module.

The location of the attention module application was chosen between the Backbone and Neck to ensure that the pre-trained weights of the backbone feature extraction network could continue to be used and to avoid training the model from scratch. The location of the attention module deployed between the backbone network and Neck is the set of three feature maps (feature1, feature2, feature3) provided to Neck by the backbone network.

**FIGURE 5:**
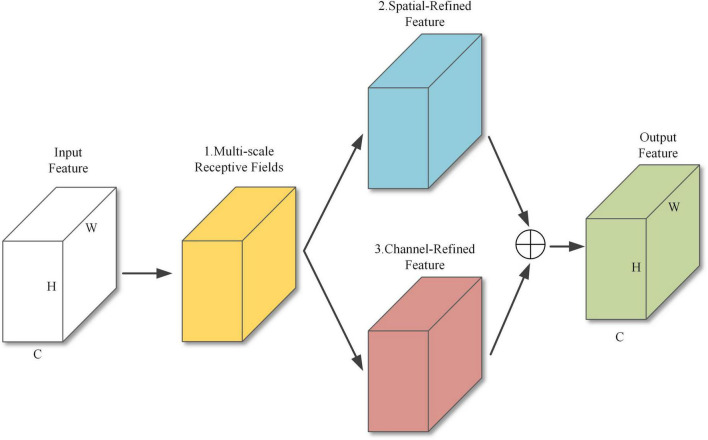
MFFAM structure.

The structure of MFFAM is shown in Figure 5 and contains 3 main sub-modules. Sub-module 1 is a multi-scale receptive field structure, and sub-modules 2 and 3 are two branches of a parallel spatial-channel attention module.

#### Multi-scale receptive field structure

**FIGURE 6:**
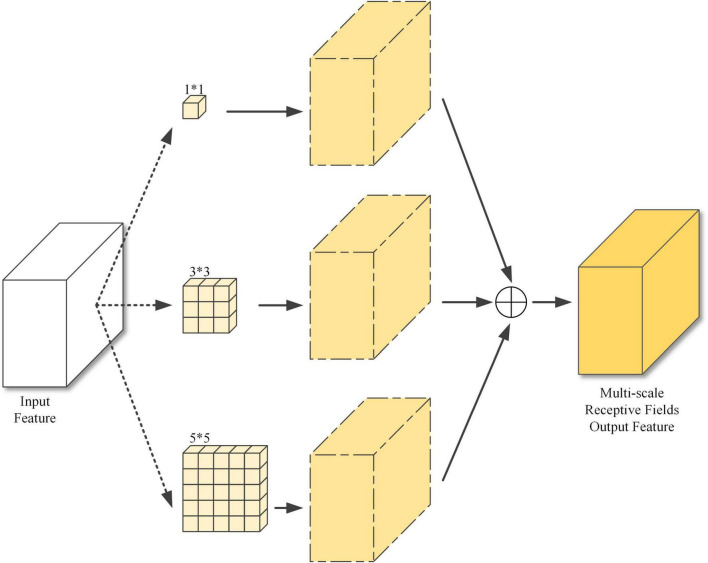
Sub-module 1: Multi-scale receptive field structure.

Feature1 (52*52), feature2 (26*26), and feature3 (13*13) are all in the deeper layers of the model, where the image information is highly extracted and compressed, and the features are very abstract, hence the need to enhance the image representations. Sub-module 1 enables different scales of perception through 3 different sizes of receptive fields and finally feature fusion to obtain the output features, as shown in Figure 6.

The large, medium, and small resolution feature maps are mainly used to detect small, medium, and large objects. If too small a receptive field is used for large-resolution feature maps, only local information is passed to the attention module, reducing the detection accuracy of small objects. If too large a receptive field is used for small-resolution feature maps, then there will be information from other objects passing into the attention module, which will make training more difficult and increase the time needed for convergence. Therefore, the receptive fields of 3*3 and 5*5 were used for the feature1, three scales of 1*1, 3*3, and 5*5 for feature2, and 1*1 and 3*3 for feature3.

#### Parallel spatial-channel attention structure

**FIGURE 7:**
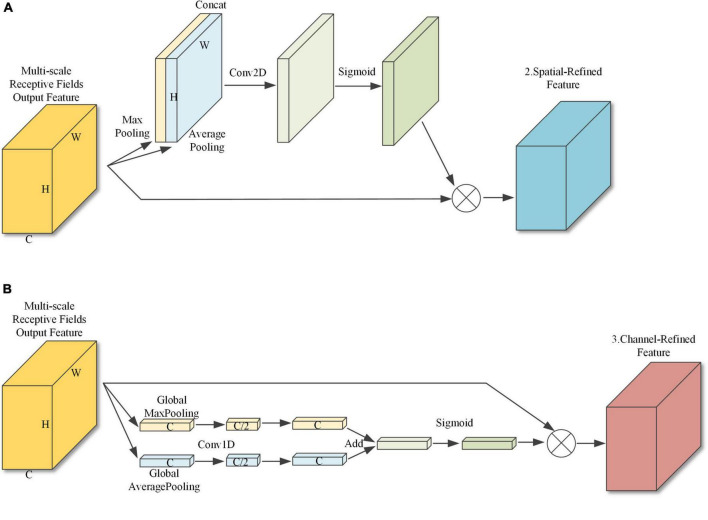
Parallel attention module. **(A)** Sub-module 2: Spatial attention module. **(B)** Sub-module 3: Channel attention module.

With a traditional CBAM attention module, channel attention is first applied to the input features to obtain the channel modulation features and then spatial attention is applied to obtain the final output features. However, as spatial attention is applied to the channel modulation features, the effect of the spatial attention module is influenced by the channel attention module. Therefore, this paper uses a parallel application method. The parallel attention module is shown in Figure 7. Sub-module 2, shown in Figure 7A, compresses the spatial information by merging the pixel information at the same position on each channel, then adjusts the number of channels with a convolution layer, and after Sigmoid activation performs feature fusion with the original input features. Sub-module 3, shown in Figure 7B, performs global average pooling and global maximum pooling on each feature map to compress the channel information, and then uses one-dimensional convolution for feature learning, with the convolution kernel size set to 7 to ensure the cross-channel information interaction rate.

## Results

### Datasets and operating environments

There are few publicly available steel surface defect inspection datasets in China. The commonly used one is the NEU surface defect database (Song and Yan, 2013; He et al., 2019; Bao et al., 2021) collected and collated by Mr. Song Kechen from Northeastern University, which contains six types of typical strip steel surface defects: Crazing (Cr), Inclusion (In), Patches (Pa), Pitted Surface (PS), Rolled-in Scale (RS) and Scratches (Sc). Each image is an intercept of the area containing the defect from the photograph taken. Each image in the dataset has a raw resolution of 200*200 pixels, for a total of 1800 images. As it is an intercepted shot, the relative area of the defect in the image is large, and it is not difficult to locate as well as classify them. A reduction in the difficulty of detection caused by human factors can make training less effective. The final model has reduced detection accuracy for small objects in practical applications. To improve the suitability of the dataset, the images need to be enhanced. This can be achieved by using image stitching (four 200*200 images stitched together into one 400*400 image). However, the surface defect detection task in strip steel is foreground localization and identification against a simple background. In the images acquired by the inspection system, a single-tone flat strip surface occupies a large percentage of the area, in addition to the object to be inspected. Therefore, by applying a solid color fill around the image, it is possible to restore the inspection scene for strip steel. Also, Mosaic was used during the training process. The solid color fill adds a simple background to the dataset image. The random scaling and stitching of images used by Mosaic will simultaneously increase the complexity of the foreground and background and improve the robustness of the model during the training process. Increasing the resolution by simply stitching the images together has no advantage when it comes to training. The fill pixels are the average grayscale of the whole image and the size after the filling is 416*416. At this point, the average area share of the object is reduced to 1/4 of the original data set. Randomly divide the training, validation, and test sets according to the ratio of 6:2:2.

The definition of a small object differs in different situations. Currently, the distinction is mainly based on absolute pixel values or relative area ratios. For example, MS COCO defines an object as small object with color image pixels less than or equal to 32*32. In this paper, objects with pixels less than or equal to 40*40 (relative area less than or equal to 0.925%) are defined as small objects. The vast majority of Crazing and a small number of Inclusions were found to be small objects during the pre-processing process. In the actual detection process, some of the Inclusions that are labeled as a whole target are divided into smaller segments. This article is based on the label and only uses Crazing as a small object.

The training GPU for this experiment was the Tesla V100S. The GPU used for the test was an NVIDIA RTX 2060 6G video memory based on Tensorflow-GPU version 2.3.0 and the CPU was an Intel Core i5-9400@2.9GHz with DDR4 2667MHz 16G+16G memory. We designed the experiments using the same dataset, training settings, and pre-training weights. Dropout is introduced during the training process to reduce overfitting. The number of epochs is set to 500, the batch size is set to 64, the initial learning rate is set to 0.01, and the learning rate is automatically adjusted by the cosine annealing method during the training process. Equation (4) is the cosine annealing method.


(4) $$lr=lr_{min}+\frac{1}{2}(lr_{max}-lr_{min})(1+cos(\frac{E}{E_{i}}\pi ))$$

where *lr*_*max*_ denotes the maximum learning rate, set as the initial learning rate; *lr*_*min*_ denotes the minimum learning rate, set as 0.0001; *E* denotes the total number of epochs and *E*_*i*_ denotes the current epoch rounds.

To verify the lightweight design and the optimization of the attention module, Recall(R), Precision(P), F1-score(F1), mean Average Precision (mAP), and Frames Per Second (FPS) were used as evaluation metrics. The calculation is done by selecting a cross-ratio IoU threshold of 0.5, and an IoU > 0.5 is considered a successful detection of the object. Equation (5) is the formula for Recall, Equation (6) is the formula for Precision, and Equation (7) is the formula for the F1 score. The mAP is calculated using the equation from VOC2010 onward as shown in Equation (8).


(5) $$R=\frac{TP}{\left(TP+FN\right)}$$

(6) $$P=\frac{TP}{\left(TP+FP\right)}$$

(7) $$F1=2(\frac{1}{\frac{1}{P}+\frac{1}{R}})=2\frac{P*R}{P+R}$$

(8) $$mAP=\frac{1}{n}\sum \int_{0}^{1}P(R)dR$$

In this paper, the *n* is 6.

### Comparison of results and analysis

#### Ablation study

To verify the effectiveness of the various improvement strategies proposed, a series of ablation experiments were carried out. The network models were trained using the same settings in the framework of experiments on the same platform. The impact of the proposed improvement modules on the performance of the network model, as well as the improvement effect of multiple modules acting together, is verified separately in the test set. The results are shown in Table 1.

**TABLE 1:** Ablation study results for enhanced YOLOX.

No.	Light-weighting	Attention module	Parameters	Model size	GFLOPs	FPS	mAP
0			505.16M	19.8MB	6.400	70.14	76.29
1	√		295.01M	12.0MB	4.086	81.34 (+11.2)	76.19 (–0.1)
2		√	511.58M	20.1 MB	6.432	67.07 (–3.07)	81.44 (+5.15)
3	√	√	301.41M	12.3MB	4.118	82.87 (+12.73)	81.21 (+4.92)

1) Scenario 1 introduces the L-CSP module in the backbone of the baseline network model. The use of the L-CSP module has had a very good compression effect on the volume of the model. The amount of computation was reduced by 36.16%, the detection framerate improved by 11.2 FPS, while the mAP decreased by only 0.1%. The experimental results demonstrate the effectiveness of the proposed lightweight design in this paper.
2) Scenario 2 deploys the MFFAM between the Backbone and Neck in the manner described in 3.2.1 based on the baseline network model. The results show that the method was able to achieve an mAP of 81.44% on the NEU-DET dataset. Compared to the baseline model, the mAP has improved by 5.15%. At the same time, the volume of calculations has increased by only 0.5%, with minimal fluctuations. The experimental results validate that MFFAM can improve the performance of the model at a fraction of the cost.
3) Scenario 3 is the deployment of MFFAM based on the lightweight model of Scenario 1. The combination of these two optimizations resulted in an mAP of 81.21 and a detection frame rate of 82.87FPS. Compared to the baseline model, the improvement was 4.92% and 12.73 FPS, respectively. The results show that the model with the introduction of both enhancements has better detection performance.

**FIGURE 8:**
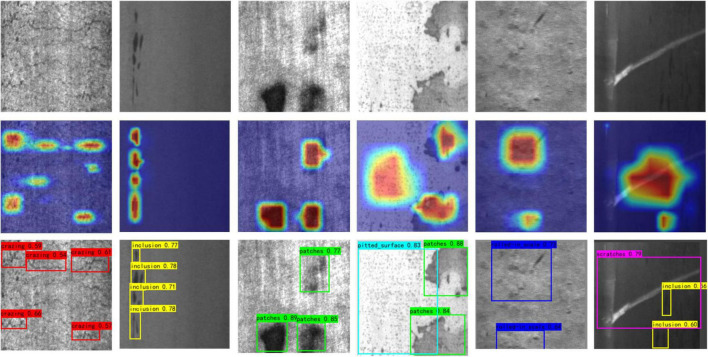
Original images, heat maps and detection images.

The model shown in Scenario 3 is the optimal model for this paper. The network model of Scenario 3 was used to detect randomly selected images of steel surface defects, and the intercepted original image, heat map, and detection results are shown in Figure 8.

The specific P, R and F1 scores for Crazing (Cr), Inclusion (In), Patches (Pa), Pitted Surface (PS), Rolled-in Scale (RS) and Scratches (Sc) of each scenario are shown in Table 2.

**TABLE 2:** Comparison of P, R, and F1 for different improvements of YOLOX.

No.	Cr			In			Pa			PS			Rs			Sc		
						
	P	R	F1	P	R	F1	P	R	F1	P	R	F1	P	R	F1	P	R	F1
0	48.2	35.1	0.41	84.6	81.9	0.83	84.4	87.7	0.86	82.4	77.8	0.80	69.7	62.2	0.66	88.6	90.7	0.90
1	69.4	35.5	0.25	78.8	68.5	0.73	88.5	82.1	0.85	94.6	92.1	0.93	73.9	51.5	0.61	87.0	83.3	0.85
2	71.8	37.9	0.50	85.5	70.7	0.77	90.5	79.8	0.85	89.2	86.8	0.88	63.3	57.6	0.60	88.9	83.3	0.86
3	68.5	40.2	0.64	84.2	75.0	0.79	83.6	82.6	0.88	94.6	92.1	0.93	73.1	57.6	0.64	82.6	87.5	0.85

**FIGURE 9:**
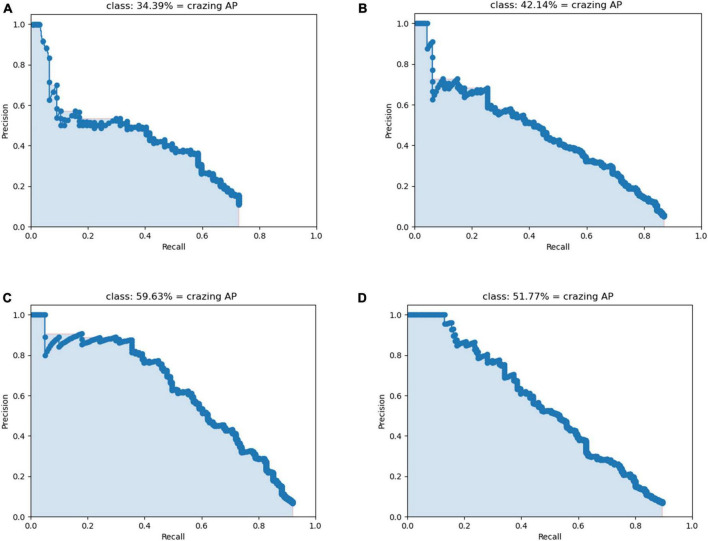
P-R diagram of Crazing. **(A)** Scenario 0. **(B)** Scenario 1. **(C)** Scenario 2. **(D)** Scenario 3.

The data in Table 2 shows that the P, R, and F1 scores of the Crazing improved after the introduction of the attention module. Precision improved by 20.2, indicating a significant improvement in performance in target localization among the detected targets. However, the Recall values are still not high compared to the other defects, suggesting that there is still a high rate of missed detection for Crazing, which may stem from the lack of distinctive features of this type of target. The P-R diagram for Crazing is shown in Figure 9, where (A)-(D) correspond to scenarios 0-3 in the ablation experiment, respectively. It can be visualized from Figure 9 that the attention module proposed in this paper is of great help in improving the detection accuracy of Crazing.

#### Performance comparison with best-in-class deep network models

To fully evaluate the superior performance of the enhanced YOLOX proposed in this study for the detection of surface defects in strip steel, the best deep network models of their kind in the last two years were selected for performance comparison. The models chosen were all network models using the NEU-DET dataset. A comparison of performance is shown in Table 3.

**TABLE 3:** Performance comparison for different network models on dataset NEU-DET.

No.	References	Model	mAP	FPS
1	Zhang et al., 2022	YOLOv5	95.5	27.03
2	Li and Wang, 2021	YOLOv5	72.2	
3	Cheng and Yu, 2020	RetinaNet	78.25	12.2
4	Sun et al., 2022	YOLOv5	85.5	27.71
5	Yuan et al., 2022	ResNet	97.22	
6	Liang et al., 2022	ResNet	98.88	
7	Lu et al., 2021	ResNet	98.05	7.69
8	Liu Y. et al., 2021	YOLOv4	91.13	26.39
9	Li X. et al., 2022	YOLOv5	76.8	43.3
10	Our Paper 2022	YOLOX	81.21	82.87

As can be seen in Table 3, there is a significant decrease in the model’s mAP when the researchers want to increase the detection frame rate. The increase in frame rate is relatively limited. In fact, most strip steel production lines require much higher detection speeds, except for certain special steels that are produced with strict quality requirements. We, therefore, started with a lightweight design to improve detection speed. On top of the lightweight modules, we use the attention module to improve the detection accuracy as much as possible. Although the accuracy in this paper is not very high compared to models that focus on accuracy. However, the comprehensive performance and practicality of the proposed model in this paper have significant advantages.

## Conclusion

A lightweight defect detection network incorporating a multi-scale feature fusion attention module has been proposed. To improve the speed of surface defect detection, this paper constructs a lightweight L-CSP structure based on the YOLOX object detection network, reducing the number of model parameters by 40.33% and the computational effort by 35.66% without affecting the detection accuracy. To improve the detection accuracy, a multi-scale feature fusion attention module MFFAM is proposed. The module uses different scales of receptive fields for feature maps of different resolutions, which are then passed in parallel into the spatial-channel attention modules, significantly improving the detection accuracy of small area defects as well as the overall mAP. To simulate a production environment, the 200*200 images in the original NEU-DET dataset are filled with pixels to 416*416 in this paper, and the relative area of the defective object is reduced to 1/4 of the original size. The results show that the overall model in this paper is able to improve the mAP from 76.29 to 81.21% on the populated dataset and improve the detection speed from 70.14 to 82.87 FPS. Compared to other defect detection models, the proposed method improves detection speed while ensuring accuracy, which is beneficial to practical applications.

## Data availability statement

The raw data supporting the conclusions of this article will be made available by the authors, without undue reservation.

## Author contributions

RWu: conceptualization, methodology, writing—original draft preparation, and writing—review and editing. NL and HL: data curation, formal analysis, and visualization. RWa and NG: software, resources, and investigation. FZ: validation and supervision. All authors have read and agreed to the published version of the manuscript.
